# Human ILC1s target leukemia stem cells and control development of AML

**DOI:** 10.21203/rs.3.rs-2319959/v1

**Published:** 2023-01-10

**Authors:** Michael Caligiuri, Zhenlong Li, Rui Ma, Hejun Tang, Jianying Zhang, Guido Marcucci, Jianhua Yu

**Affiliations:** City of Hope National Medical Center; City of Hope National Medical Center; City of Hope National Medical Center; City of Hope National Medical Center; City of Hope National Medical Center; City of Hope Medical Center; City of Hope National Medical Center

## Abstract

Innate lymphocytes can mediate cancer immunosurveillance and protect against disease. We have demonstrated that mouse type I innate lymphoid cells (ILC1s) can contribute to controlling the growth of acute myeloid leukemia (AML). However, the functional roles of human ILC1s in AML remain largely undefined. Here, we found that the ILC1s in patients with AML are impaired while a high expression of the ILC1 gene signature is associated with better overall survival in AML. By directly interacting with leukemia stem cells (LSCs), human ILC1s can eliminate LSCs via production of IFNγ and block LSC differentiation into M2 macrophage-like, leukemia-supporting cells through TNF. Collectively, these effects converge to limit leukemogenesis *in vivo*. We also identified Lin^−^CD127^+^CD161^−^CRTH2^−^CD117^−^ cells as the human ILC1 subset. The use of umbilical cord blood (UCB) CD34^+^ hematopoietic stem cells to generate CD161^−^ ILC1s could allow for a readily available supply of ILC1s to be produced for human adoptive transfer studies. Together, our findings provide evidence that targeting human ILC1s may be a promising therapeutic approach for prolongation of disease-free survival in AML.

Acute myeloid leukemia (AML) is a devastating disease and the median 5-year survival is 40–45% for patients younger than age 65 treated with standard chemotherapy^1^. Although allogeneic stem cell transplantation has shown to be curative in some cases, the treatment-related mortality and the risk for disease relapse due to the possible persistence of leukemia stem cells (LSCs) remain relatively high. Therefore, safer and more effective novel therapeutic approaches are needed to improve clinical outcomes of patients with AML. The innate lymphoid cell (ILC) plays a critical role in mediating immune responses, regulating tissue homeostasis, and inflammation^2–7^. We recently reported that mouse ILC1s contribute to the control of AML by eliminating LSCs and inhibiting their differentiation into myeloid blasts^8^. Functional impairment of mouse ILC1s in AML leads to the outgrowth of LSCs and disease relapse^8^. However, the potent anticancer properties and mechanisms of human ILC1s in AML remain to be further explored. Our study suggests that human ILC1s are clinically relevant in halting progression of AML and have the potential to be manufactured ex vivo for the successful treatment of AML.

## Results And Discussion

**Figure 1:**
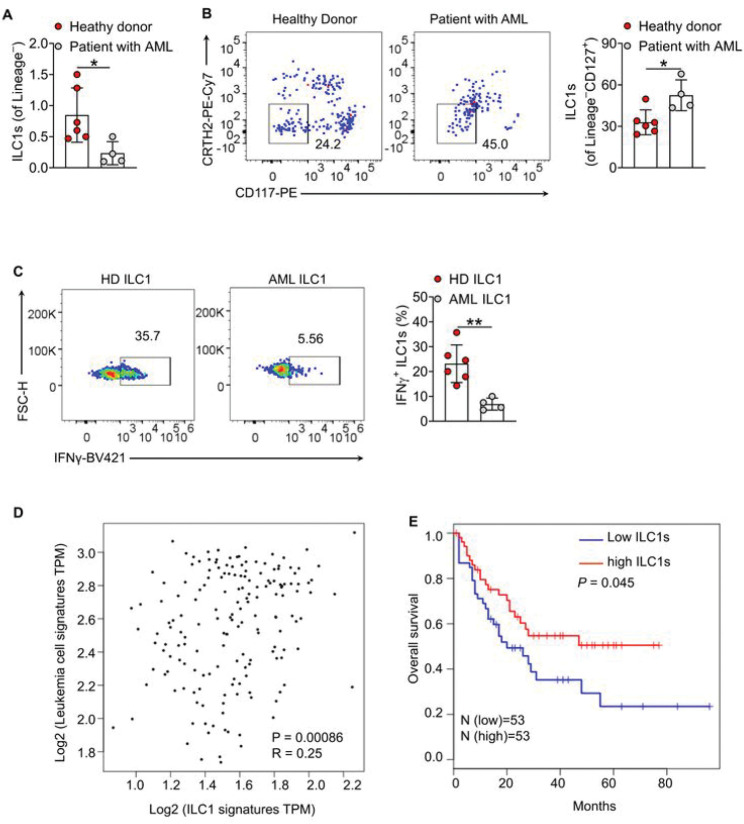
ILC1s are impaired and their levels correlate with favorable clinical outcomes in patients with AML. (**A**) Statistics of the percentages of total ILC1s among lineage negative cells in the peripheral blood of healthy donors and patients with AML. (**B**) Representative flow cytometry plots of the percentages of (left) and statistics of the percentages (right) of ILC1s among total ILCs (gated on Lin^−^CD127^+^) in the peripheral blood of healthy donors and patients with AML. (**C**) Human mononuclear cells isolated from the peripheral blood of healthy donors (HD ILC1) and patients with AML (AML ILC1) were stimulated with 1 μl leukocyte activation cocktail for 4 h, and then the intracellular staining was performed using anti-IFNγ. Representative flow cytometry plots of (left) and statistics of (right) the IFNγ^+^ ILC1s. (**D**) Correlation analyses on ILC1 signature (*IFNG, TNFA, TRAIL, CD49A, NKP46*, and *RORA*) and leukemia cell (leukemia blasts / LSCs) signature (*CD34/CD33, CD133, CD7*, and *CD13*) in TCGA-LAML cohort. Two-tailed Pearson correlation test. (**E**) Survival analyses based on ILC1 signature in TCGA-LAML individual cohort (n = 53). Log-rank Mantel-Cox test. Data from three experiments were pooled. Data are presented as mean ± S.D.; *P* values were calculated by one-way ANOVA. *P < 0.05; **P < 0.01.

We have reported that the higher IFNγ levels produced by ILC1s isolated from mice can induce apoptosis of LSCs^8^. However, most of the mechanistic results were demonstrated in mouse ILC1s. Whether ILC1s control LSC fate also in the human AML remains largely undefined. Following analysis of ILC1s in the blood of patients with AML at disease onset, we observed a highly significant reduction of total ILC1s among lineage-negative cells (Lin^−^, defined as depletion of CD3, CD4, CD8, CD14, CD15, CD16, CD19, CD20, CD33, CD34, CD203c, FceRI, and CD56 positive cells) relative to healthy donors (Fig. 1A). However, among total ILCs (defined as Lin^−^CD127^+^) in the same patient population, there was a significant enrichment of the ILC1 subset relative to healthy donors (Fig. 1B). Further, the IFNγ^+^ functional ILC1s were significantly reduced in the patients with AML compared to healthy donors (Fig. 1C). Together, these data suggest that ILC1s in patients with new onset AML are impaired in their frequency among Lin^−^ cells and in their production of IFNγ. Using The Cancer Genome Atlas (TCGA) database, we identified a significant positive correlation between ILC1s and leukemia blast and/or LSC signatures (R = 0.25, p = 0.00086) (Fig. 1D). Analysis of 53 AML cases from TCGA showed that AML patients with high ILC1 gene signature had a significantly prolonged overall survival compared to AML patients with a low ILC1 gene signature (Fig. 1E). Collectively, these data suggest that the functional roles of human ILC1s become dysregulated in the context of AML, and a high level of human ILC1s correlates with more favorable clinical outcomes in AML.

**Figure 2:**
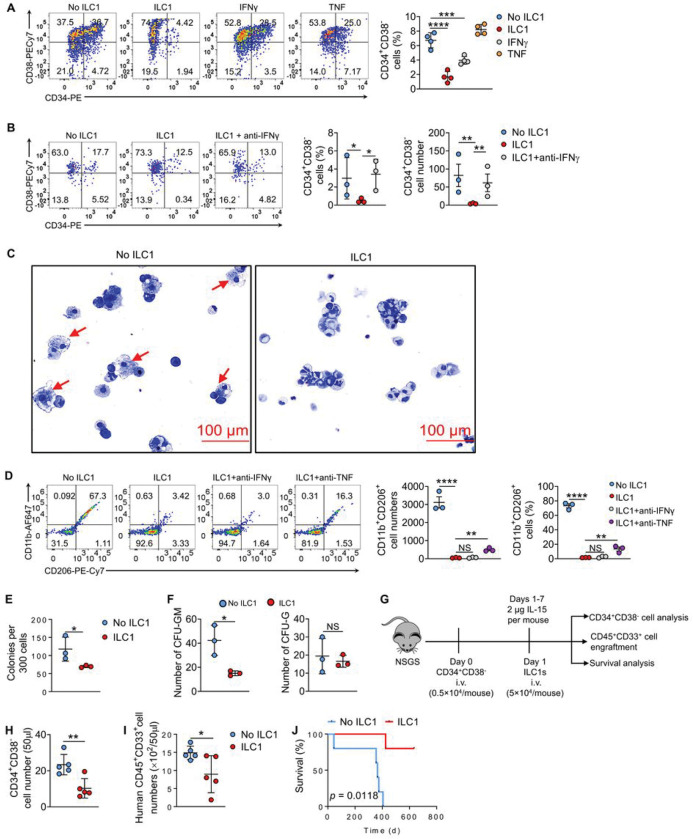
At a lower density, human ILC1s target human LSCs and block their differentiation into M2-like macrophages. **(A)** Human LSCs from the blood of patients with AML were co-cultured with or without human ILC1s in the presence or absence of human recombinant IFNγ (10 ng/ml) and TNF (10 ng/ml) for 3 days. Representative flow cytometry plots (left) and statistics (right) of the percentage of CD34^+^CD38^−^ cells (n = 4). (**B**) Human LSCs were co-cultured with or without human ILC1s in the presence or absence of human anti-IFNγ (10 μg/ml) for 3 days. Representative ow cytometry plots (left) and statistics (right) of the percentage and absolute cell number of CD34^+^CD38^−^ cells (n = 3). (**C**) Human LSCs were co-cultured with or without human ILC1s for 3 days. Representative images of Wright-Giemsa staining of cells (20× magnification, scale bar, 100 μm, n = 3). (**D**) Human LSCs were co-cultured with or without human ILC1s for 3 days. Representative flow cytometry plots (left) and statistics of the absolute cell number (middle) and percentage (right) of CD11b^+^CD206^+^ cells (n = 3). (**E** and **F**) Human LSCs were co-cultured with or without human ILC1s for 3 days. Total colony-forming cells (E) and colony-forming myeloid progenitors (CFU-GM and CFU-G; F) were counted at each round of plating (n = 3). (**G**) Schematic of the design and procedures for (H-G). 0.5×10^4^ human CD34^+^CD38^−^ cells were i.v. injected into NSG-SGM3 mice on day 0. One day later, 5×10^4^ human ILC1s were i.v. injected into these mice. Three weeks later, the numbers of human CD45^+^CD33^+^ cells and CD34^+^CD38^−^ cells were analyzed. (**H**) Statistics of the absolute cell number of CD34^+^CD38^−^ cells in the blood of NSG-SGM3 mice. (**I**) Statistics of the absolute cell number of CD45^+^CD33^+^ cells in the blood of NSG-SGM3 mice. (J) Survival of the mice transplanted with LSCs and treated with or without ILC1s (n = 5). Survival data were analyzed by Kaplan–Meier survival analysis and log-rank test. All non-survival data are shown as mean ± SD. *P* values were calculated by either one-way ANOVA or student’s t test. *P<0.05; **P<0.01; ***P<0.001; ****P<0.0001; NS, not significant.

To investigate the impact of human ILC1s (defined as Lin^−^CD127^+^CD161^+^CRTH2^−^CD117^−^, hereafter referred to as CD161^+^ ILC1s or ILC1s) on human LSCs (CD34^+^CD38^−^ cells), we isolated LSCs from the peripheral blood of patients with AML and cocultured them with human ILC1s isolated from the peripheral blood of healthy donors at a ratio of one ILC1 to four LSCs (1:4), or with recombinant human (rh) IFNγ or rhTNF for 3 days. We observed that compared to the control group of LSCs alone, coculture of LSCs with ILC1s or with rhIFNγ, but not with rhTNF, significantly decreased the CD34^+^CD38^−^ cell fraction (Fig. 2A). The percentage and absolute number of CD34^+^CD38^−^ cells were reversed in an identical coculture of ILC1s with LSCs that was treated with anti-IFNγ and compared to coculture of ILC1s with LSCs alone (Fig. 2B), indicating that ILC1−produced IFNγ eliminated LSCs. To understand whether ILC1s can affect the differentiation of LSCs into the non-LSC fractions, we sorted CD34^+^CD38^−^ cells and cocultured them with ILC1s at the 1:4 ratio of ILC1s to LSCs for 3 days. By Wright-Giemsa staining, we observed that ILC1s blocked the differentiation of CD34^+^CD38^−^ cells into macrophage-like leukemia-supporting cells (Fig. 2C) that were previously reported to support the growth of leukemic cells rather than inhibiting them^9–11^. Flow cytometry of the differentiated cells showed that some express CD11b and CD206 (CD11b^+^CD206^+^) with an M2 phenotype^9^, and the population, equivalent to macrophage-like leukemia-supporting cells, was found to be significantly decreased in the presence of ILC1s (Fig. 2D). When TNF neutralizing antibody was added to the culture of ILC1s with LSCs, the ability of ILC1s to block the differentiation of LSCs decreased modestly but significantly, as evidenced by partial recovery of the otherwise decreased CD11b^+^CD206^+^ population in the presence of the neutralizing TNF antibody and ILC1s (Fig. 2D). A similar effect was not seen in the presence of neutralizing IFNg. This indicates that ILC1-derived TNF rather than IFNγ at least partially contributes to suppressing the differentiation of LSCs into M2 macrophage-like leukemia-supporting cells. The colony-forming unit (CFU) assay further confirmed that ILC1s impede the differentiation of human LSCs into leukemia-supporting M2 macrophages, as indicated by decreased numbers of total colonies and granulocyte-macrophage progenitor (CFU-GM) colonies after coculture with ILC1s compared to culture without ILC1s, while there was no significant difference in the number of granulocyte (CFU-G) colonies (Fig. 2E and 2F). Collectively, these data indicate that both IFNγ and TNF produced by ILC1s contribute to the control of human CD34^+^CD38^−^ LSC development *in vitro*, the former eliminated it while the latter blocked its differentiation into leukemia-supporting M2 macrophages. We also performed the *in vivo* transplantation experiment, in which human CD34^+^CD38^−^ cells and human ILC1s were co-injected i.v. into NOD.Cg-*Prkdc*^*scid*^ *Il2rg*^*tm1Wjl*^ Tg(CMV-IL3,CSF2,KITLG)1Eav/MloySzJ (NSG-SGM3) mice expressing human hIL3, hGM-CSF (CSF2) and hSCF (KITLG), three cytokines that support the stable engraftment of myeloid lineages^12^. On each of days 1–7, the mice received an intraperitoneally (i.p.) injection of human IL-15. We found that the injection of human ILC1s from healthy individuals reduced the LSC engraftment into mice and suppressed the progression of AML, as evidenced by the significantly decreased number of CD45^+^CD33^+^ blast cells, the significantly decreased number of CD34^+^CD38^−^ LSCs, and the significantly prolonged survival of mice, all compared to mice that did not receive injection of ILC1s (Fig. 2G–2J). These data suggest that human ILC1s can suppress the differentiation and development of human LSCs *in vivo*. Taken together, these findings demonstrate that ILC1s play a positive role against AML and provide a rationale for using ILC1s as a cellular-based therapy to prolong disease-free survival in AML.

ILC1s have been considered CD161-expressing cells. However, there is no evidence that isolation and *ex vivo* expansion of Lin^−^CD161^+^ ILC1s from AML patients or healthy donors results in a homogeneous population of ILC1s. Through extensive flow cytometric analyses, we found that ILC1s were heterogeneous in the peripheral blood of humans using ILC1-specific surface markers (Lin, CD127, CRTH2, and CD117) that were reported previously^13,14^ and a combination of CD161 antibody (Extended data Fig. 1A). The heterogeneous ILC1s included the conventional CD161^+^ ILC1s and Lin^−^CD127^+^CD161^−^CRTH2^−^CD117^−^cells (hereafter referred to as CD161^−^ ILC1s). In our hands, the percentage of CD161^−^ ILC1s was higher than CD161^+^ ILC1s among total ILCs isolated from the blood of healthy donors (Extended data Fig. 1A). We further expanded the total ILCs on either OP9-DL1 or DL4-transfected OP9 (hereafter referred to as DL1 and DL4) stromal cells in the presence of IL-2, IL-7, and IL-15 and found that there were far fewer CD161^+^ ILC1s and a far greater number of CD161^−^ ILC1s regardless of whether they were co-cultured with DL1 or DL4 stromal cells (Extended data Fig. 1B–1E). The CD161^−^ ILC1s were further identified by the expression of IFNγ, Eomes, and T-bet (Extended data Fig. 1F and 1G), indicative of an ILC1 lineage. Using the same method recently reported for derived ILCs from umbilical cord blood (UCB) CD34^+^ hematopoietic stem cells (HSCs)^15^, we obtained ILC1s that were almost entirely CD161^−^, as demonstrated by the phenotype, including the expression of IFNγ, Eomes, and T-bet (Extended data Fig. 1H–1J). A nearly 700-fold increase from UCB CD34^+^ cells to CD161^−^ ILC1s (> 97% purity) was observed on day 28 (Extended data Fig. 1K). The cytotoxicity against LSCs was comparable between CD161^−^ ILC1s differentiated from UCB CD34^+^ HSCs and the conventional CD161^+^ ILC1s isolated from peripheral blood mononuclear cells (PBMCs; Extended data Fig. 1L). Because ILC1s are a minute population in PBMCs and it is very difficult to expand and produce adequate numbers of fresh total ILCs *ex vivo* for therapeutic use, these results pave the way for developing a novel adoptive cell transfer therapy, using UCB CD34^+^ HSCs to generate a readily available, allogeneic, and off-the-shelf supply of CD161^−^ ILC1s for treating diseases such as AML.

## Methods

### Human samples

Peripheral blood samples were obtained from healthy donors and patients with AML at the City of Hope National Medical Center (COHNMC). Mononuclear cells were isolated using Ficoll separation. LSCs were sorted using a BD FACSAria^™^ Fusion (BD Biosciences). ILCs were isolated using EasySep^™^ Human Pan-ILC Enrichment Kit (STEMCELL) or were sorted using a BD FACSAria^™^ Fusion. Umbilical cord blood leukopaks were obtained from StemCyte, Inc (Baldwin Park, Californai). CD34^+^ cells were isolated using the CD34 MicroBead Kit (Miltenyi Biotec). All healthy donors and patients with AML signed an informed consent form. Sample acquisition was approved by the Institutional Review Board at the COHNMC.

### Cells and cell culture

OP9-DL1 and DL4 were maintained in DMEM GlutaMAX media supplemented with 20% FBS, 100 U/ml penicillin, 0.1 mg/ml streptomycin, and 50 μM β-Mercaptoethanol. Human LSCs were cultured in StemSpan^™^ Serum-Free Expansion Medium II with penicillin (100 U/mL), streptomycin (100 mg/mL), stem cell factor (SCF, 20 ng/ml), thrombopoietin (TPO, 20 ng/ml), Flt3-L (20 ng/ml), IL-3 (10 ng/ml), and IL-6 (10 ng/ml). Human ILC1s were cultured in MEMα GlutaMAX media. Media was supplemented with 10% human AB serum, IL-2 (500 IU/ml), IL-7 (20 ng/ml), and IL-15 (20 ng/ml). Cultures were incubated at 37°C in a humidified atmosphere of 5% CO2.

### Flow cytometry

ILC1s from human peripheral blood were identified using surface staining with a live/dead cell viability cell staining kit and the following monoclonal antibodies: lineage (FITC-anti-CD3, anti-CD4, anti-CD8, anti-CD14, anti-CD15, anti-CD16, anti-CD19, anti-CD20, anti-CD33, anti-CD34, anti-CD203c, anti-FceRI), CD56 (FITC, AF700 or BV421-anti-CD56), CD127 (APC-anti-CD127), CRTH2 (PE-Cy7-anti-CRTH2), and c-Kit (BV711-anti-c-Kit). Human LSCs were identified by lineage (FITC-anti-CD2, anti-CD3, anti-CD4, anti-CD8, anti-CD14, anti-CD19, anti-CD20, anti-CD11b, anti-CD56, and anti-CD235a), CD45 (BV510-anti-CD45), CD34 (PE-anti-CD34), and CD38 (BV605- or PE-Cy7-anti-CD38). To examine intracellular cytokine production, intracellular staining for IFN-γ, Eomes, and T-bet was performed using a Fix/Perm kit (eBiosciences), followed by staining with a BV421-anti-IFN-γ antibody, an APC-anti-T-bet antibody, or BUV395-Eomes, respectively. All analyses were performed on a Fortessa X-20 flow cytometer (BD Biosciences) and sorting was performed using a BD FACSAria^™^ Fusion.

### Survival analysis and correlation analysis

Survival analysis and pairwise correlation analysis of gene expression signatures were performed using GEPIA2^16^, based on TCGA and GTEx databases. There were 53 AML tumor samples included in the survival analysis. We established gene expression signatures for ILC1s and leukemia blasts and/or LSCs based on previous human studies^13,17–20^. For each signature gene set, AML cohort was divided into high and low expression groups by median value (50% cut-off). ILC1: *IFNG, TNFA, TRAIL, CD49A, NKP46*, and *RORA*. Leukemia cell (leukemia blasts / LSCs): *CD34, CD33, CD133, CD7*, and *CD13*. Survival analyses were performed with log-rank Mantel-Cox test. Pairwise correlation analyses of gene expression signatures were performed with two-tailed Pearson correlation test.

### LSCs and ILC1s in vitro co-culture assay

Human LSCs from patients with AML were labeled with 5 mM CellTrace^™^ Violet dye (CTV) and co-cultured with or without ILC1s (Lin^−^CD127^+^CRTH2^−^CD117^−^) isolated from the peripheral blood of healthy donors in the presence of human IL-12 (10 ng/ml) and IL-15 (100 ng/ml). For coculture assays with cytokines and antibodies, human LSCs were cocultured with or without IFNγ (10 ng/ml), TNF (10 ng/ml), anti-IFNγ antibody (10 μg/ml), or anti-TNF (10 μg/ml) antibody. For all co-culture assays, after three days of co-culture, cells were harvested and analyzed using flow cytometry. Annexin V and 7-amino-actinomycin D (7-AAD, BD Biosciences) were used to identify dead cells following the manufacturers’ instructions.

### In vitro colony-forming unit assay

Human LSCs were isolated from the blood of patients with AML and were cocultured with or without ILC1s isolated from the blood of healthy donors for 3 days. Cells were then plated into human methylcellulose complete media (R&D, HSC003) supplied with recombinant human SCF (50 ng/ml), human recombinant IL-3 (10 ng/ml), IL-6 (10 ng/ml), recombinant human GM-CSF (10 ng/ml), and recombinant human EPO (3 IU/ml). Cultures were incubated at 37°C in a humidified atmosphere of 5% CO2 for 10–14 days. Colony numbers were counted using a microscope (Zeiss AxioCam 702).

### In vivo LSC transplantation assay

For the human LSC engraftment experiment, 0.5×10^4^ human LSCs were isolated from peripheral blood or bone marrow cells of patients with AML and then transplanted via tail vein injection into sublethally irradiated (200 cGy) 6–8-week-old NOD.Cg-*Prkdc*^*scid*^*Il2rg*^*tm1Wjl*^ Tg(CMV-IL3,CSF2,KITLG)1Eav/MloySzJ (NSG-SGM3) purchased from the Jackson laboratory. All mice were maintained by the Animal Resource Center of COH. Mouse care and experimental procedures were performed in accordance with federal guidelines and protocols approved by the Institutional Animal Care and Use Committee at City of Hope. One day later, 5×10^4^ human ILC1s isolated from the peripheral blood of human healthy donors were injected via the tail vein into these mice. Recombinant human IL-15 (2 μg/mouse) was intraperitoneally injected into recipient mice daily for 7 days. Engraftment of human CD45^+^CD33^+^ and CD34^+^CD38^−^ cells in the blood of mice was monitored at 3 weeks.

### In vitro ILC1 induction from umbilical cord blood (UCB) CD34^+^ hematopoietic stem cells

CD34^+^ cells from UCBs were sorted by FACS and were plated onto approximately 80–90% confluent OP9-DL1 or OP9-DL4 stromal cell monolayers in MEMα GlutaMAX media (Thermo Fisher Scientific). Media was supplemented with 10% human AB serum, and SCF (20 ng/ml), Flt3-L (20 ng/ml), IL-7 (20 ng/ml), IL-15 (20 ng/ml), and IL-3 (5 ng/ml) at the first week. One week later, IL-3 cytokine was removed from the media. Two weeks later, Flt3-L was reduced to 5 ng/ml. Media and cytokines were refreshed every 3–4 days by replacing half of the media containing 1× concentration of cytokines. At the end of weeks 1, 2, and 3, cells were replated onto fresh OP9-DL1 or OP9-DL4 onto six-well plates.

### Statistical analysis

For continuous endpoints, Student’s *t* test was used to compare two independent conditions and one-way ANOVA models were used to compare three or more independent conditions. For survival data, survival functions were estimated by the Kaplan–Meier method and compared by log-rank tests. All tests were two-sided. *P* values were adjusted for multiple comparisons by Holm’s procedure. Data are presented as mean ± SD. Prism software v.8 (GraphPad, CA, USA) and SAS v.9.4 (SAS Institute. NC, USA) were used to perform statistical analyses. The p-values are represented as: * <0.05, ** <0.01, *** <0.001, and **** <0.0001
